# Common hepatic bile duct partial transection: a rare consequence of blunt abdominal trauma. A case report and brief narrative review

**DOI:** 10.1093/jscr/rjaf101

**Published:** 2025-03-05

**Authors:** Mohamed Abdelgawad, Luis Fernandez, Diana Wu, Justin Sacks, Amit Mori, Jason Murry, Sally Abdelgawad, Omar Kamel, Faris Mahjoub

**Affiliations:** Department of Surgery, University of Texas Health Science Center, UT Health East Texas, 11937 US-Highway 271, Tyler, TX 75708, United States; Department of Surgery, Division of Trauma Surgery/Surgical Critical Care, The University of Texas Health Science Center, UT Health East Texas, 1000 S Beckham Ave, Tyler, TX 75701, United States; The University of Texas-Tyler School of Medicine Bill Barrett Inaugural Endowed Chair in Trauma Surgery, Trauma Wound Care, UT Health East, 11937 US-Highway 271, Tyler, TX 75708, United States; Department of Surgery, University of Texas Medical Branch, 301 University Blvd, Galveston, TX 77555, United States; Carle Illinois College of Medicine, University of Illinois, 506 S Mathews Ave, Urbana-Champaign, IL 61801, United States; Department of Radiology, UT Health East Texas, 701 Olympic Plaza Cir, Tyler, TX 75701, United States; Department of Gastroenterology, UT Health East Texas Digestive Disease Center, UT Health East Texas, 700 Olympic Plaza Cir, Tyler, TX 75701, United States; Division of Trauma Surgery/Surgical Critical Care, The University of Texas Health Science Center, UT Health East Texas, 11937 US-Highway 271, Tyler, TX 75708, United States; Department of Oral and Maxillofacial Surgery, Virginia Commonwealth University, 910 W Franklin St, Richmond, VA 23284, United States; St. George’s University School of Medicine, Old Town Mill, True Blue Street, St. George’s, Grenada, West Indies; School of Health Sciences, Oakland University, 2200 N Squirrel Rd, Rochester Hills, MI 48309, United States

**Keywords:** blunt abdominal injury, blunt extrahepatic injury, bile duct injury, delayed complication, case report, extrahepatic biliary injury, blunt abdominal trauma, delayed traumatic injury

## Abstract

Extrahepatic biliary injuries are rare in the settings of blunt abdominal trauma and often have delayed presentation. These findings of an isolated distal common hepatic duct injury secondary to blunt trauma may provide insight into the considerations in the progression of care especially as surgical intervention is not always required. In many cases, adopting a multidisciplinary approach involving interventional radiology and gastroenterology can provide more comprehensive care and lead to better outcomes. We hereby present a rare case and literature review of a common hepatic bile duct partial transection as a consequence of a blunt abdominal trauma. Highlighting the collaborative nature of the approach and emphasizing the potential benefits for patients.

## Introduction

Non-iatrogenic traumatic blunt extrahepatic biliary injuries are reported as low as 1 in 10 500 cases [1]. This occurrence is low when compared to non-iatrogenic traumatic blunt intrahepatic biliary injuries (2.8%–7.4%) [2]. Blunt extrahepatic biliary injuries are often missed on initial presentation. There are three common locations where blunt extrahepatic ductal injury may occur: the origin of the left hepatic duct, the bifurcation of the hepatic ducts, and the pancreaticoduodenal junction [3]. These anatomical areas are more susceptible and are at increased risk of injury due to excessive shearing forces. While there is no specific guideline for blunt extrahepatic biliary injuries, the American Association for the Surgery of Trauma (AAST) has developed a grading scale for extrahepatic biliary duct injury, and Losanoff and Kjossev have published a classification system for blunt gallbladder (GB) injuries. These references have helped provide initial understandings and approaches to these rare cases especially as blunt injuries can have delayed clinical appearances and delayed treatment may be optimal as well [4, 5]. Operative repair is best completed within 7 days (early) or after 6 weeks (delayed) of injury [6, 7]. See Supplementary Table 1.

## Clinical case

A 55-year-old male with a history of chronic obstructive pulmonary disease, smoking, and colorectal cancer, on chemotherapy, presented as a level 1 trauma activation, after a motor vehicle crash. The patient had a right hip deformity and was intubated in the field. On arrival, he was hypotensive and tachycardic that responded to fluid resuscitation. A-line and central lines were placed. FAST exam was negative; no penetrating injuries were noted. A chest x-ray showed bilateral pneumothoraces and bilateral chest tubes were placed. A subsequent pelvic X-ray showed a right hip fracture, which was reduced. Computed tomography with intravenous contrast of the neck, chest, abdomen, and pelvis suggested bilateral grade 1 carotid injury, (this was negative on subsequent angiography); free intra-abdominal air with thickening at the duodenojejunal junction; a pancreatic injury with contrast extravasation; and a right pelvic fracture which was repaired by orthopedic surgery during his stay.

### Initial operation /damage control surgery

**Figure 1:**
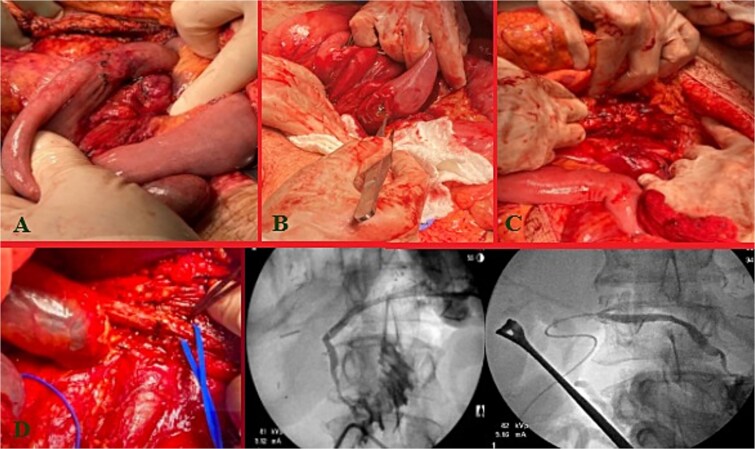
Index operation. A: Transverse colon hematoma. B: Duodenal (D2) injury. C: Pancreatic head and tail vascular injury. D: Common bile duct and intraoperative cholangiogram through the common bile duct and cystic duct.

Massive transfusion protocol was initiated, and he was taken to the operating room for an emergent exploratory celiotomy. Upon entrance into the abdominal cavity, a 10 cm hematoma, along the transverse colon consistent with a mesenteric arterial hemorrhage, was identified and rapidly controlled (Fig. 1A). A Cattell-Braasch maneuver was employed, mobilizing the right colon, and a Kocher maneuver to fully explore the duodenum, revealing a AAST Grade II D2 injury that was repaired in 2 layers (Fig. 1B). The pancreatic head and tail had AAST Grade II injuries with multiple arterial bleeds that were controlled with suture ligation and argon beam coagulation (Fig. 1C).

On initial inspection, the GB infundibulum was contused, and there was bile staining along the porta hepatis. The common bile duct (CBD) was identified and appeared grossly normal. However, due to the proximity of the pancreatic head injury and bile staining, we performed an intraoperative cholangiogram (IOC) through a mid-portion CBD needle ductotomy, to spare the GB as a potential conduit if a biliary repair was required. The initial IOC did not reveal an injury to the CBD and did not demonstrate extravasation of contrast (Fig. 1D). The needle ductotomy was closed with a running 3–0 Monocryl stitch. As we were concluding the damage control laparotomy a persistent, mild, bile staining within and along the porta hepatis, was noted. The possibility of a more proximal extrahepatic bile duct injury was raised as the common bile needle ductotomy closure remained intact.

Further inspection of the GB and proximal extra biliary ductal system revealed a large GB bed hematoma and partial avulsion of the GB (AAST Grade 3). Due to the risk of delayed GB ischemia, a cholecystectomy, and an additional IOC via the cystic duct this time was performed to rule out a more proximal ductal injury and a possible bile leak from the prior IOC distal needle ductotomy. The more proximal and distal IOC did not reveal a bile leak, however, the more proximal ductal structures were not optimally demonstrated. No further bile staining was noted. The abdomen was irrigated, and 4 Jackson-Pratt (JP) drains and an AbThera™ Advance Open Abdomen Dressing (3 M, Saint Paul, MN, USA) were placed. The patient was stabilized and transferred to the intensive care unit (ICU) for further resuscitation, on triple antibiotics, pantoprazole, and a somatostatin drip.

### Post-operative course

**Figure 2:**
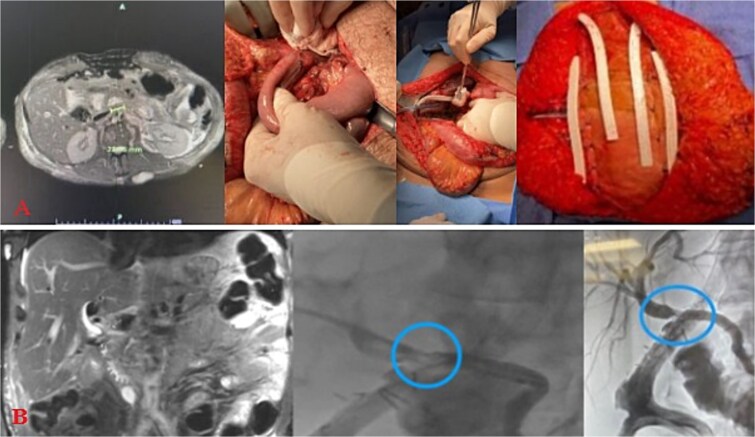
Injury sequelae and interventions. A: Re-exploration due to bilious changes. B: Delayed presentation of common hepatic duct stricture with rendezvous procedure.

The patient stayed a total of 77 days at the hospital with around 54 days spent in the ICU. He was initially in ICU care for his extensive traumatic and complicated recovery. On POD1–3 increased bilious output was noted from a JP drain. He was taken back to the operating room (OR) on POD3 for re-exploration. There was a small contained biliary collection of ~2.23 cm in the GB fossa without active bile leak noted, viable bowel, and intact duodenal repair. The patient underwent placement of a gastrostomy and feeding jejunostomy tubes, an appendectomy, and abdominal wall reconstruction with Ovitex. He returned to the OR on POD7 for frank blood noted in another JP drain where a persistent gush of blood was noted at the GB fossa. This was repaired with multiple clips and figure of 8 sutures. The abdomen was packed and the Abthera wound vac was replaced. Repeat re-exploration on POD8 noted continued hemostasis, but there remained bile near the porta hepatis and two additional JP drains were left in the region of concern. He recovered well for the consequent days with stabilized bilirubin levels (Fig. 2A). However, on POD20, it was noted that he had increasing bile output of over 1 L over 24 hours. The bile was consequently fed back through his J-tube, and on POD25, a hepatobiliary iminodiacetic acid scan (HIDA) scan failed to demonstrate the leak and an initial ERCP attempt failed secondary to a duodenal diverticula. On POD27, magnetic resonance cholangiopancreatography (MRCP) demonstrated a common hepatic duct stricture. ERCP was successful in cannulation of the ampulla on POD32 with continued lack of contrast extravasation at the distal common bile duct. On POD35, percutaneous transhepatic cholangiography drain was placed to relieve concerns of a leak. On POD54, interventional radiology and gastroenterology successfully completed a ERCP rendezvous procedure to place a biliary stent over the area of stricture. (Fig. 2B) This intervention successfully reduced the amount of bilious and overall JP output. On hospital Day 77, he was consequently discharged with all drains removed and tolerating a regular diet.

## Discussion

Blunt extrahepatic biliary injury is rare, with <1% occurrence in abdominal trauma [4]. The earliest case report regarding blunt extrahepatic biliary injuries was in 1799 when a man fell from his horse and passed away 8 weeks later with 12–15 L of bile documented in his abdomen in autopsy [8, 9]. The AAST and the World Society of Emergency Surgery Guidelines (WSES) have developed algorithmic recommendations to assist in the management of duodenal pancreatic and extrahepatic biliary trauma. However, the specific types of primary repair for extrahepatic biliary duct injuries are not discussed, and the algorithm currently lacks direct recommendations on the timing of initial or subsequent surgical intervention [10].

There are varying degrees of transection of the common hepatic duct. Incomplete transection, defined as <50% of the bile duct circumference, are generally repaired with primary ductal closure with or without T-tube drainage. Complete transections are repaired in Roux-en-Y hepaticojejunostomy (RYHJ) fashion [2, 11–13]. If these techniques fail, further diagnostic studies (e.g. HIDA scans, and MRCP) may be done and can provide definitive therapy (e.g. endoscopic retrograde cholangiopancreatography, ERCP and percutaneous drainage, PTC), particularly if the detection of these leaks is not within the recommended range for operative intervention (e.g. early repair within 48 hours compared to late repair defined as 12 weeks) [12–14].

There are generally four different methods in managing injuries at these locations ensuring adequate biliary drainage; ERCP and biliary stenting, PTC, RYHJ repair and primary closure with or without T-tube [11, 15]. It is important to note that not all of these procedures may be suitable in an acute trauma setting and are not without their inherent operative risks [12, 16]. Some emerging surgical techniques (such as using decellularized ureteral grafts to repair common bile duct defects) have had limited success in animal models [7, 13]. RYHJ, T-tube placement, biliary stents, and other exploratory surgeries, have yet to be fully studied, in the context of traumatic extrahepatic biliary injuries, to determine the optimal repair technique.

In this case, the patient uniquely was also completing chemotherapy at the time. With this risk, there are risks of developing bile duct necrosis associated with arterial-infused chemotherapy that may have contributed towards poor healing and/or increased susceptibility to a bile duct injury [17–20].

Identification and repair of extrahepatic biliary injuries remains challenging. It is important to maintain a high index of suspicion in the setting of blunt hepatobiliary trauma, and it should always be considered when a patient presents with visceral injuries consistent with excessive shearing forces. The types of therapeutic interventions are varied and are influenced by the injury pattern, the patient’s physiologic status, and associated co-morbidities. The principles of operative management in unstable patients follow the guidelines of damage control laparotomy. Optimal outcomes in patients with these types of injuries often require a tailored, and nuanced multidisciplinary approach.

## Supplementary Material

Table_1_Models_for_Classification_of_Bile_Duct_Injury_rjaf101

## Data Availability

Data sharing is not applicable to this article as no datasets were generated or analyzed during the current study.

## Permissions

All images are original pieces obtained with permission from the patient and the hospital. All tables created are compiled by the authors with original modifications made.
