# Supplementary material for: Common hepatic bile duct partial transection: a rare consequence of blunt abdominal trauma. A case report and brief narrative review
Source: J Surg Case Rep. 2025 Mar 5;2025(3):rjaf101. doi: 10.1093/jscr/rjaf101 (PMC11881695; doi:10.1093/jscr/rjaf101)
Supplement: Table_1_Models_for_Classification_of_Bile_Duct_Injury_rjaf101 [file table_1_models_for_classification_of_bile_duct_injury_rjaf101.pdf]

Classification Models for Bile Duct Injury	
Bismuth	• Developed in 1982<br>• Five types of bile duct injuries based on location in reference to the hilar structure
Siewert	• Developed in 1994<br>• Five types of bile duct injuries based upon complications from open surgeries including bile duct leaks and vessel injury
Strasberg	• Developed in 1995, modified Bismuth classification<br>• Updated to include considerations on injuries from laparoscopic surgeries; often listed along with Bismuth classifications
McMahon	• Developed in 1995<br>• Includes laparoscopic surgery considerations<br>• Injury classification based on width of bile duct injury with lacerations <25% of the diameter of the common bile duct as a minor injury and transection or lacerations >25% as a major injury
Amsterdam	• Developed in 1996<br>• Four types (A-D) of criteria that cover various levels of duct leaks with consideration of lumen obstruction and transections of the biliary tree
Neuhaus	• Developed in 2000<br>• Long-term injury pattern differences<br>• Five types (A-E) of complications including occlusions, stenosing, and tangential lesions
Stewart-Way	• Developed in 2004<br>• Four classes (I-IV) of injury that include a description of common mistakes in identifying abnormal anatomy
Sandha	• Developed in 2004<br>• Definitions of injury based on algorithmic pathways for ERCP in the setting of a bile leak and with possible findings of stones
Lau	• Developed in 2007, modification of the Mattox classification<br>• Five types (1-5) of criteria that describe injury at various anatomical levels with consideration of with/without tissue loss
Hanover	• Developed in 2007<br>• Five types (A to E) of injuries based on common complications from laparoscopic cholecystectomy including nearby vessels and bile leakage
ATOM (For Anatomy, For time off, For Mechanism)	• Developed in 2013<br>• Three primary categories addressing previous models for injury with context of the timing and mechanism including from early intraoperative to late, and from mechanical to energy-driven

Table 1: Models for Classifications of Bile Duct Injury
